# Supplementary figures and images for: Taxonomic revision of the genus Amphritea supported by genomic and in silico chemotaxonomic analyses, and the proposal of Aliamphritea gen. nov
Source: PLoS One. 2022 Aug 10;17(8):e0271174. doi: 10.1371/journal.pone.0271174 (PMC9365125; doi:10.1371/journal.pone.0271174)

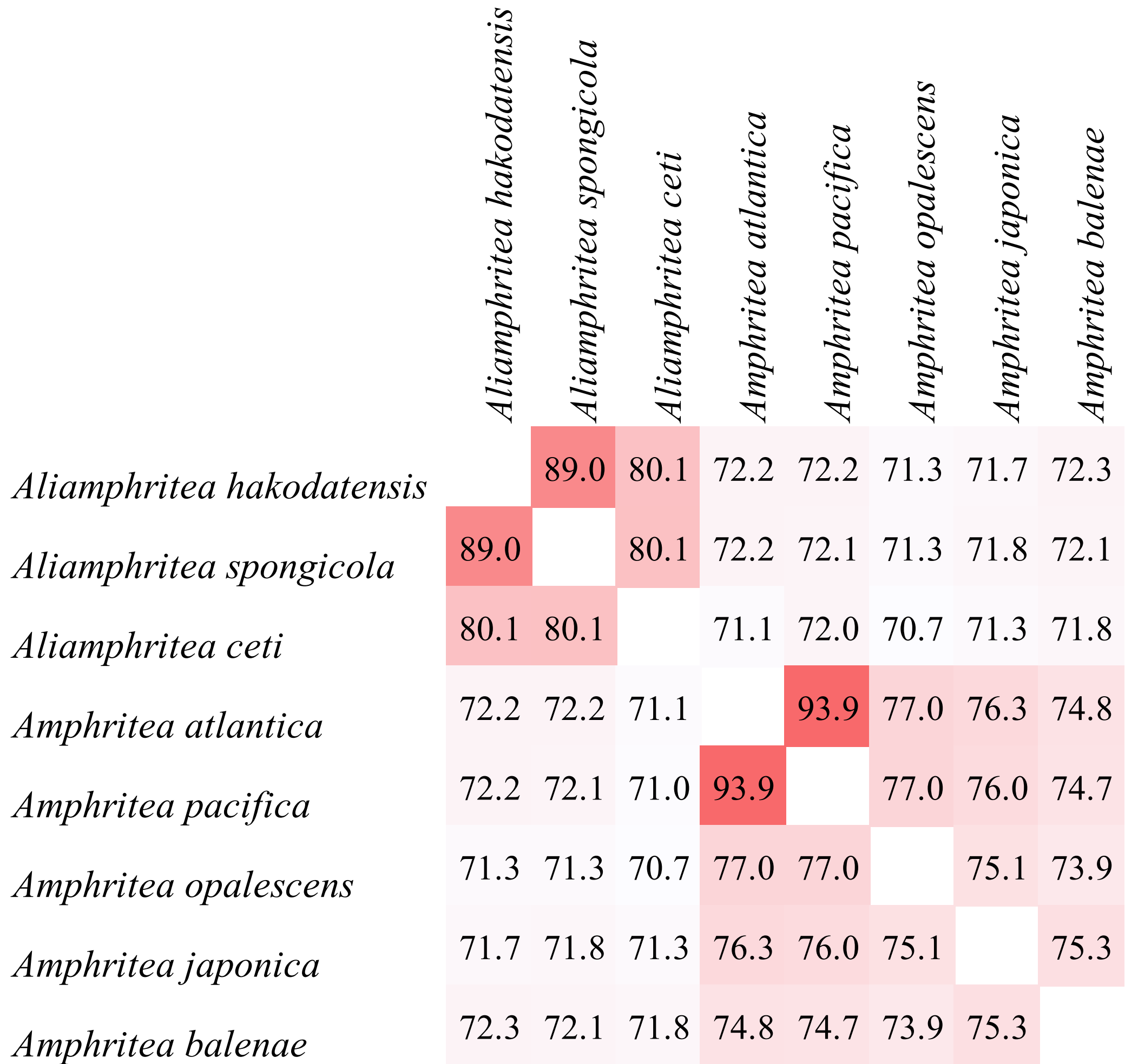

**Fig S1. Heat map representation of ANI values of *Aliamphritea* and *Amphritea* species.**

Supplement: S1 Fig — (PDF) [file pone.0271174.s001.pdf]

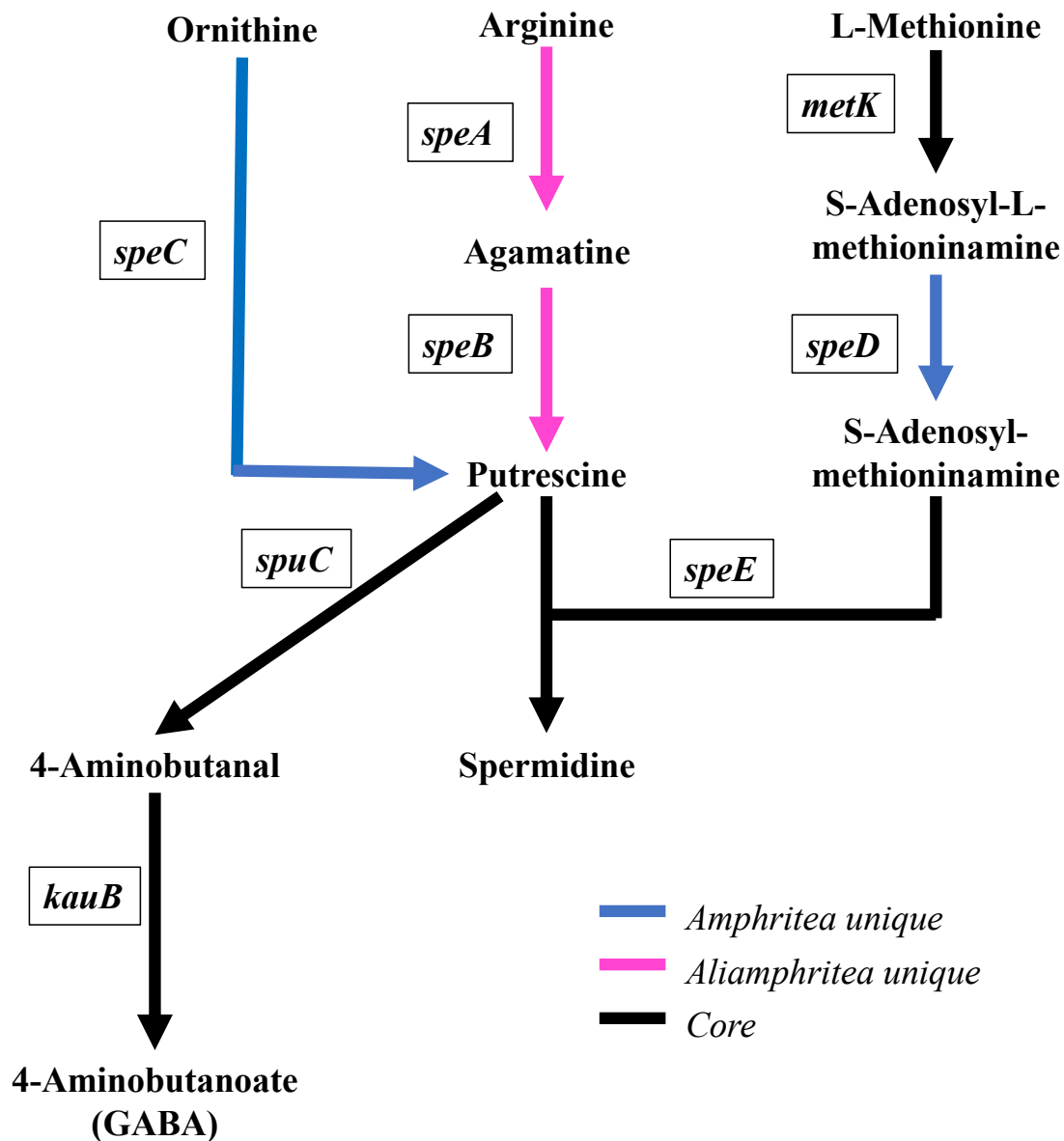

**Fig S3. Predicted polyamine metabolism pathways in *Amphritea* and *Aliamphritea* species.**

Supplement: S3 Fig — (PDF) [file pone.0271174.s003.pdf]

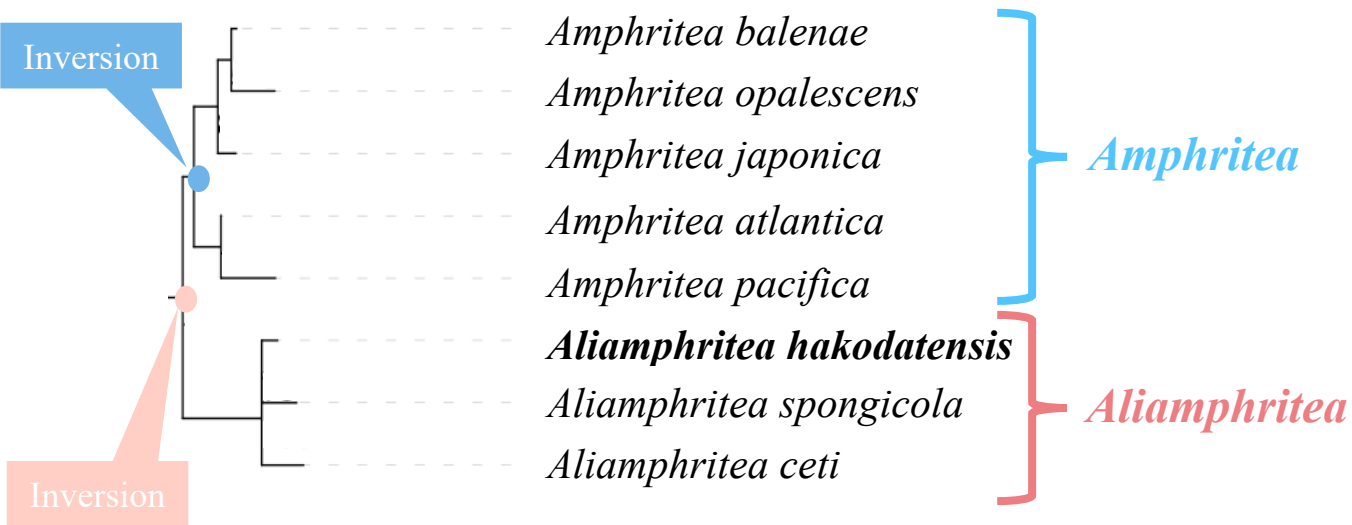

**Fig S4. Evolutionary history of *Amphritea* and *Aliamphritea* genome arrangement.**

Supplement: S4 Fig — (PDF) [file pone.0271174.s004.pdf]

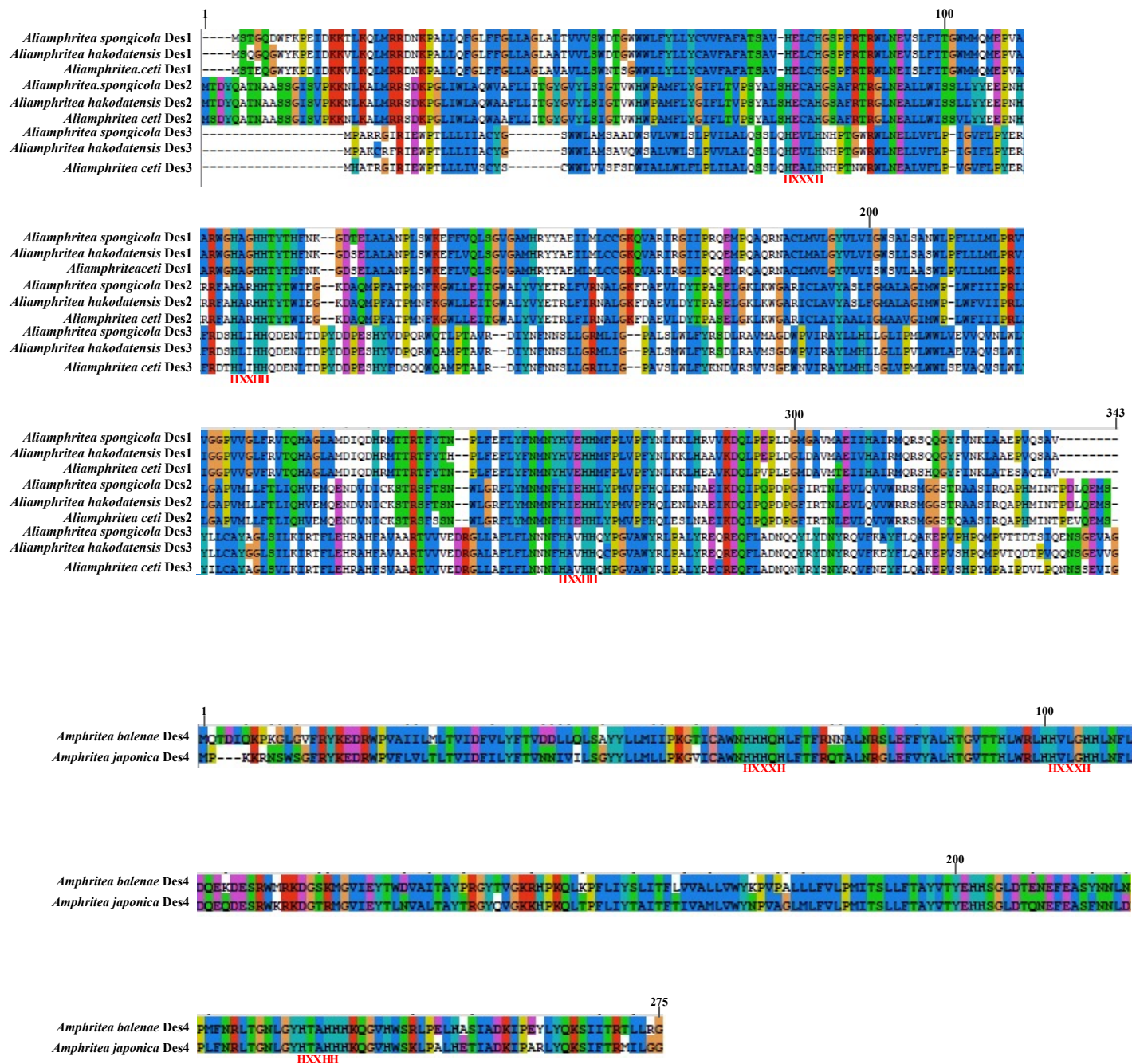

Fig S5. Amino acid sequence alignment of *Des1-4*.

Supplement: S5 Fig — (PDF) [file pone.0271174.s005.pdf]

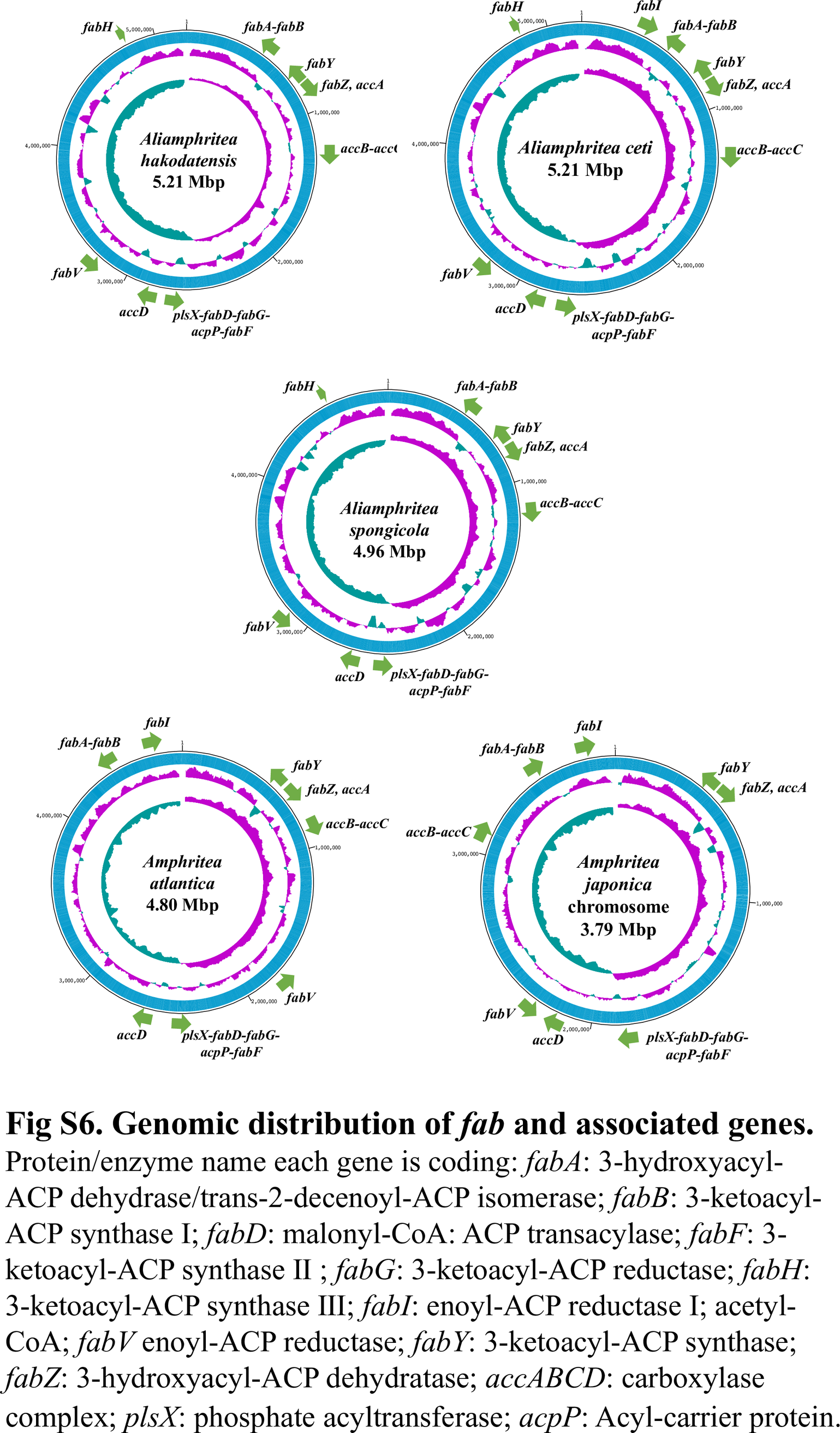

Supplement: S6 Fig — Protein/enzyme name each gene is coding: fabA: 3-hydroxyacyl-ACP dehydrase/trans-2-decenoyl-ACP isomerase; fabB: 3-ketoacyl-ACP synthase Ⅰ; fabD: malonyl-CoA: ACP transacylase; fabF: 3-ketoacyl-ACP synthase Ⅱ; fabG: 3-ketoacyl-ACP reductase; fabH: 3-ketoacyl-ACP synthase Ⅲ; fabI: enoyl-ACP reductase Ⅰ; acetyl-CoA; fabV enoyl-ACP reductase; fabY: 3-ketoacyl-ACP synthase; fabZ: 3-hydroxyacyl-ACP dehydratase; accABCD: carboxylase complex; plsX: phosphate acyltransferase; acpP: Acyl-carrier protein. (TIF) [file pone.0271174.s006.tif]

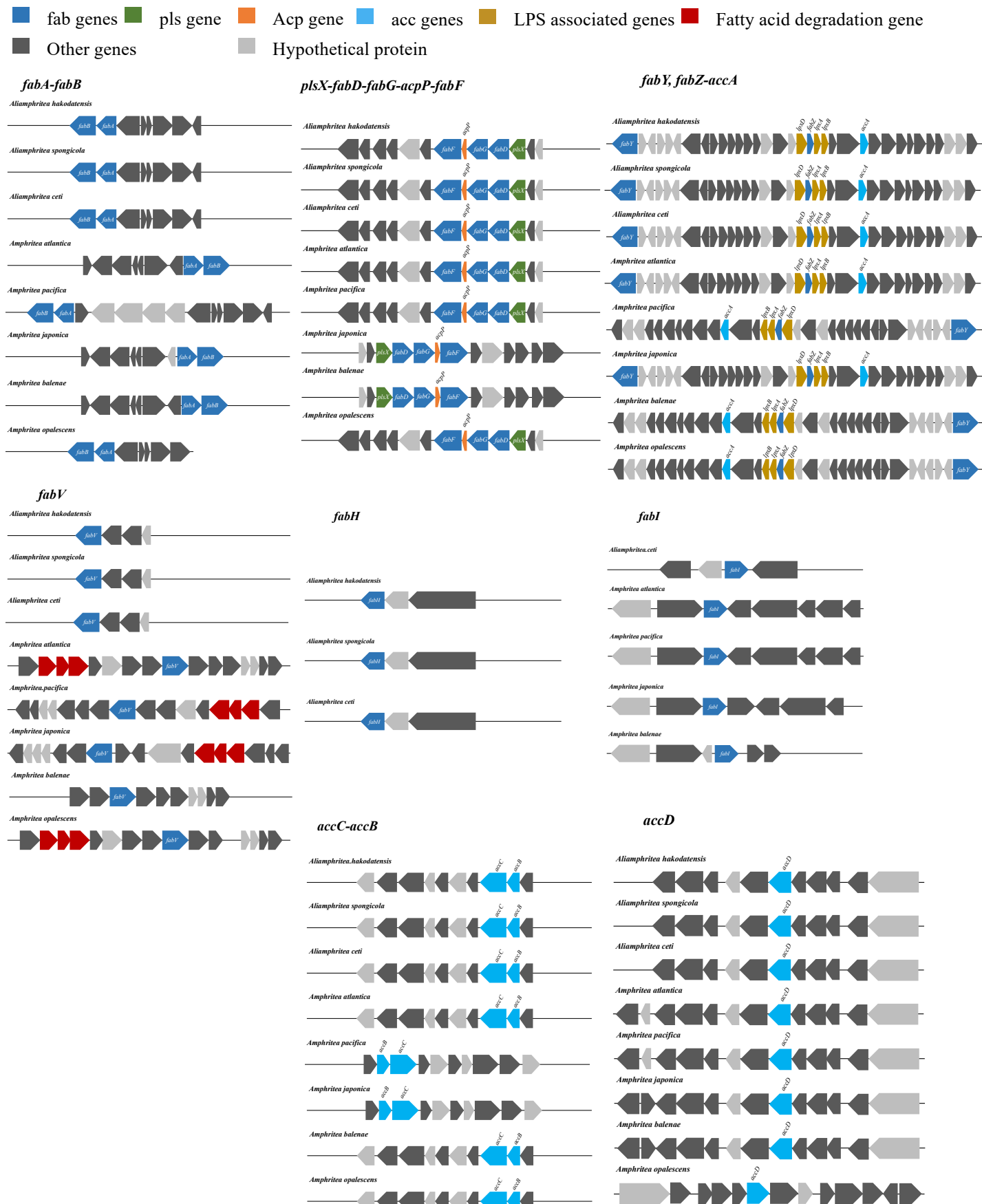

Fig S7. Genomic structure of *Amphritea* and *Aliamphritea* *fab* and associated genes.

Supplement: S7 Fig — (PDF) [file pone.0271174.s007.pdf]

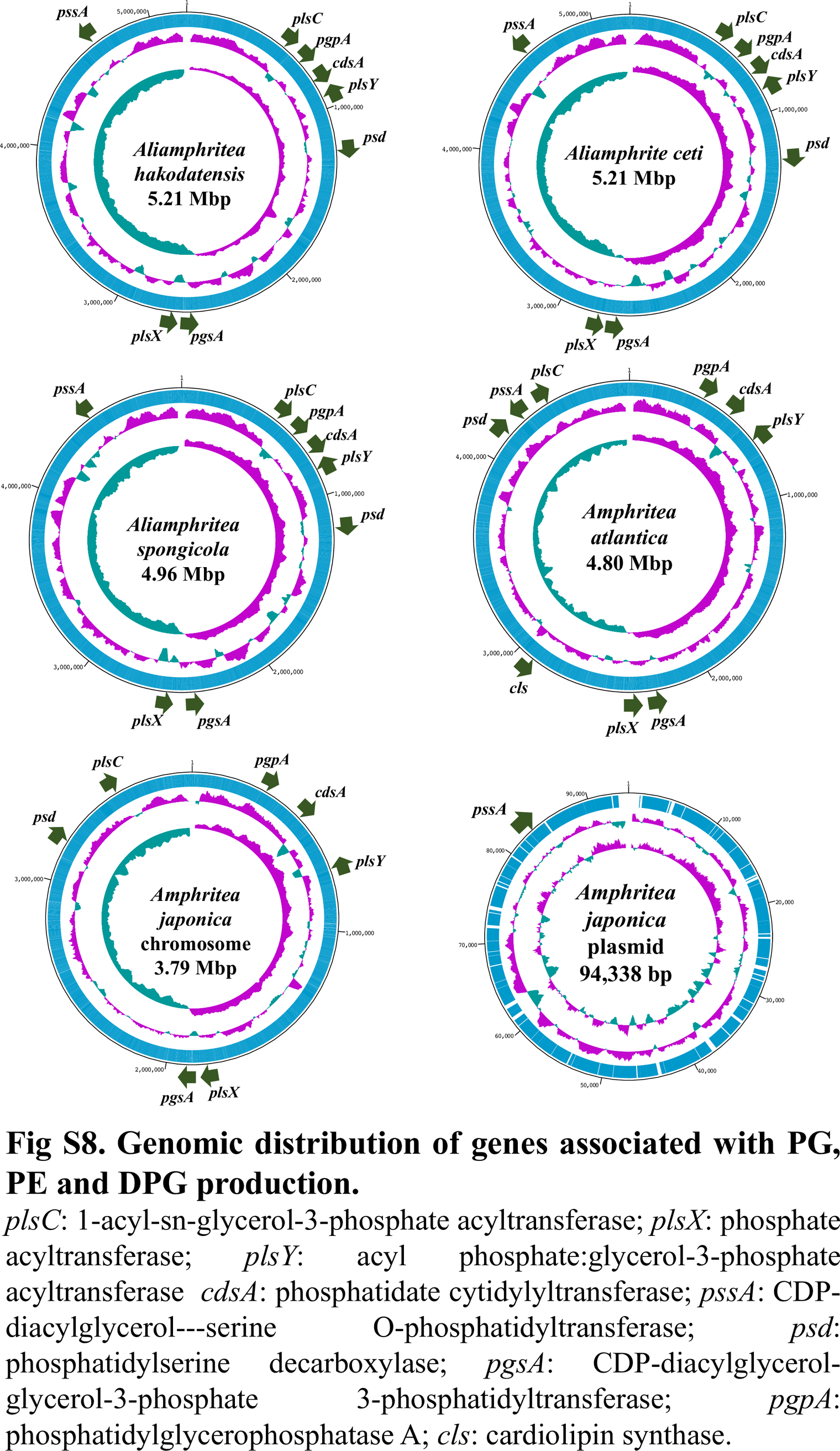

Supplement: S8 Fig — plsC: 1-acyl-sn-glycerol-3-phosphate acyltransferase; plsX: phosphate acyltransferase; plsY: acyl phosphate: glycerol-3-phosphate acyltransferase cdsA: phosphatidate cytidylyltransferase; pssA: CDP-diacylglycerol—serine O-phosphatidyl transferase; psd: phosphatidylserine decarboxylase; pgsA: CDP-diacylglycerol-glycerol-3-phosphate 3-phosphatidyltransferase; pgpA: phosphatidyl glycerophosphatase A; cls: cardiolipin synthase. (TIF) [file pone.0271174.s008.tif]

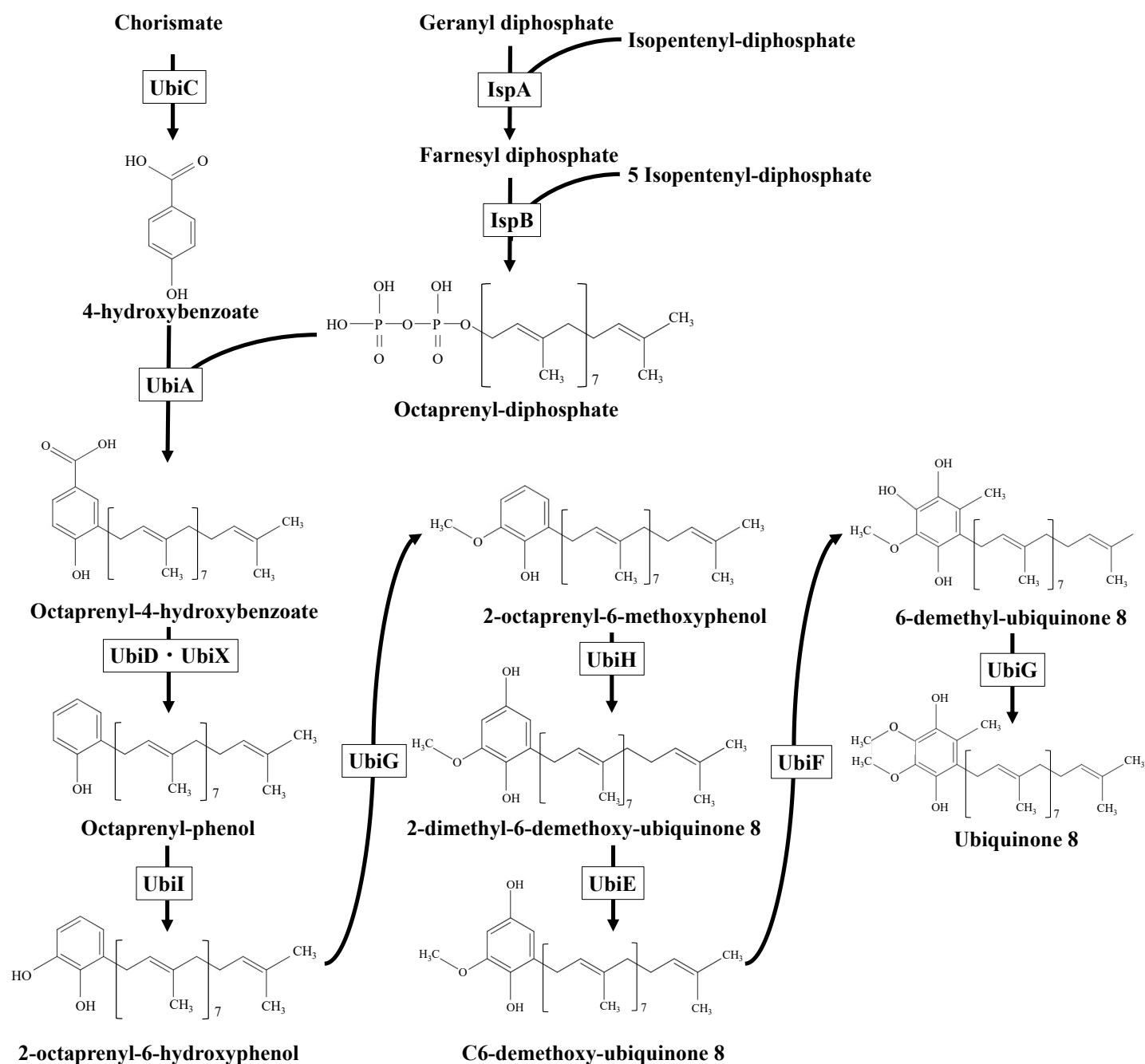

**Fig S9. Predicted Q-8 synthetic pathways in *Amphritea* and *Aliamphritea* species.**

Supplement: S9 Fig — (PDF) [file pone.0271174.s009.pdf]

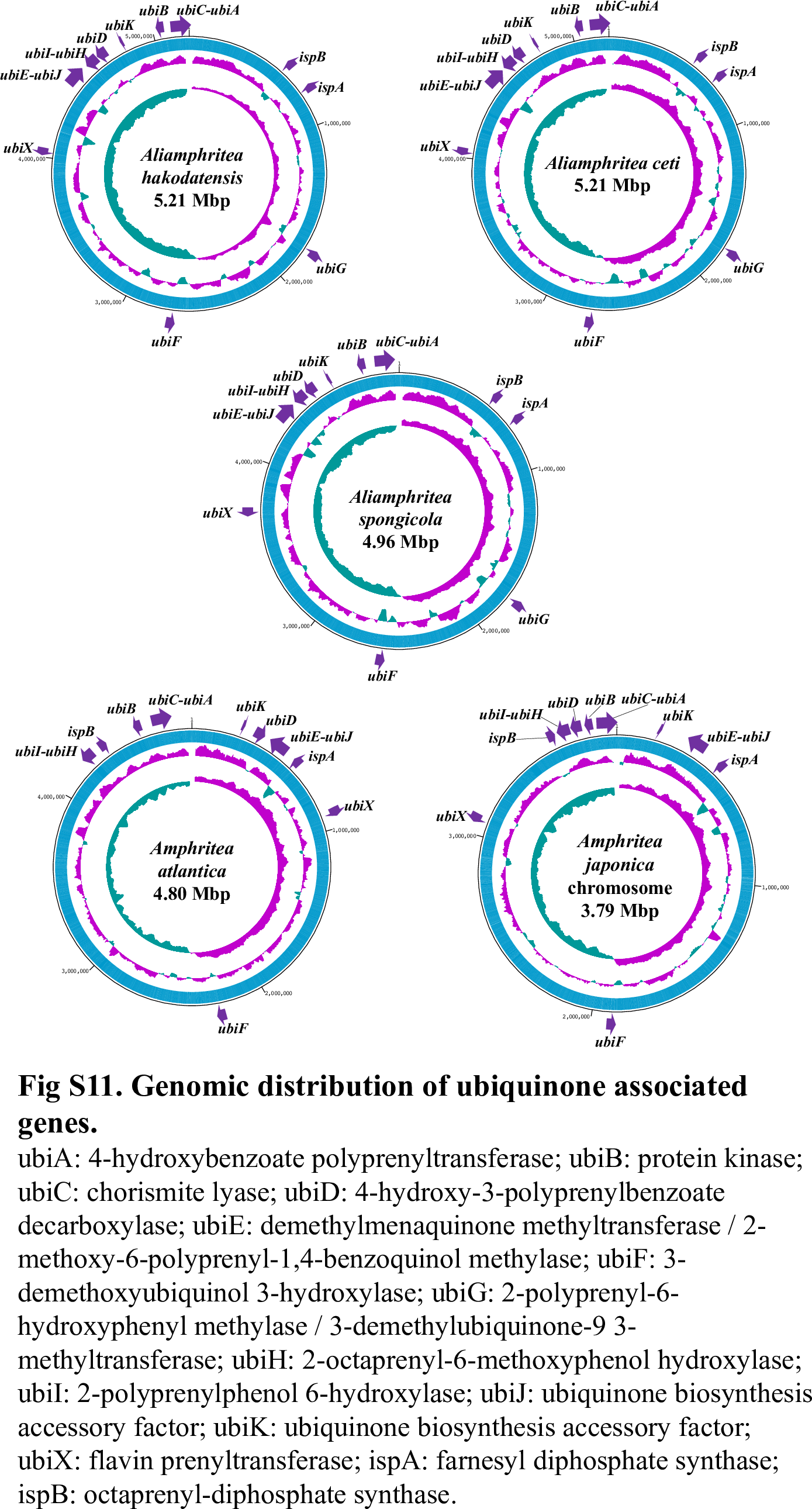

Supplement: S11 Fig — ubiA: 4-hydroxybenzoate polyprenyltransferase; ubiB: protein kinase; ubiC: chorismite lyase; ubiD: 4-hydroxy-3-polyprenylbenzoate decarboxylase; ubiE: dimethylmenaquinone methyltransferase / 2-methoxy-6-polyprenyl-1,4-benzoquinol methylase; ubiF: 3-demethoxyubiquinol 3-hydroxylase; ubiG: 2-polyprenyl-6-hydroxyphenyl methylase / 3-demethylubiquinone-9 3-methyltransferase; ubiH: 2-octaprenyl-6-methoxyphenol hydroxylase; ubiI: 2-polyprenylphenol 6-hydroxylase; ubiJ: ubiquinone biosynthesis accessory factor; ubiK: ubiquinone biosynthesis accessory factor; ubiX: flavin prenyltransferase; ispA: farnesyl diphosphate synthase; ispB: octaprenyl-diphosphate synthase. (TIF) [file pone.0271174.s011.tif]
