# Supplementary material for: Taxonomic revision of the genus Amphritea supported by genomic and in silico chemotaxonomic analyses, and the proposal of Aliamphritea gen. nov
Source: PLoS One. 2022 Aug 10;17(8):e0271174. doi: 10.1371/journal.pone.0271174 (PMC9365125; doi:10.1371/journal.pone.0271174)
Supplement: S2 Fig — The bar represents 1 μm. (PDF) [file pone.0271174.s002.pdf]

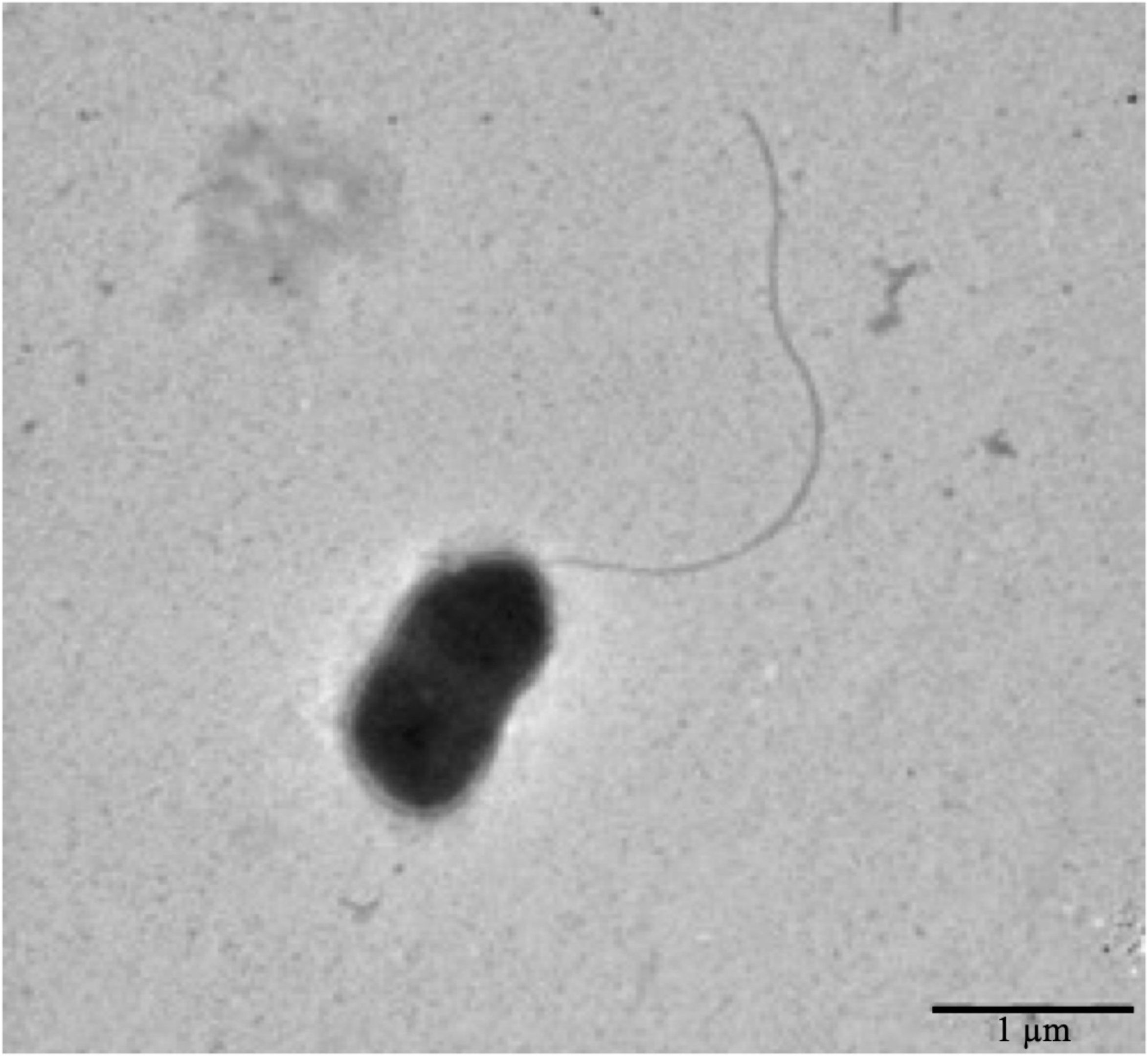

Fig S2. An electron micrograph of negatively stained *Aliamphritea hakodatensis* PT3^T cell.
The bar represents 1 μm.
