# Supplementary material for: Taxonomic revision of the genus Amphritea supported by genomic and in silico chemotaxonomic analyses, and the proposal of Aliamphritea gen. nov
Source: PLoS One. 2022 Aug 10;17(8):e0271174. doi: 10.1371/journal.pone.0271174 (PMC9365125; doi:10.1371/journal.pone.0271174)
Supplement: S1 Table — (PDF) [file pone.0271174.s012.pdf]

Table S1. Genome properties of *Aliamphritea* and *Amphritea* species

Species	Strain	Accession	Genome assemble status	Total size (genome size)	G+C content
Aliamphritea hakodatensis	PT3^T	AP025281	complete (1 chromosome)	5.21 Mb	52.2%
Aliamphritea ceti	KCTC 42154^T	AP025282	complete (1 chromosome)	5.21 Mb	47.1%
Aliamphritea spongicola	JCM 16668^T	AP025283	complete (1 chromosome)	4.96 Mb	51.5%
Amphritea atlantica	JCM 14776^T	AP025284	complete (1 chromosome)	4.80 Mb	51.1%
Amphritea japonica	JCM 14782^T	AP025761-AP025762	complete (1 chromosome, 1 plasmid)	3.88 Mb	47.5%
Amphritea balenae	JAMM1525^T	GCF_014646975.1	draft (18 contigs)	4.67 Mb	47.7%
Amphritea opalescens	ANRC-JH14^T	GCF_003957515.1	draft (46 contigs)	4.12 Mb	48.5%
Amphritea pacifica	ZJ14W^T	GCF_016924145.1	draft (282 contigs)	4.70 Mb	51.2%
