# Supplementary material for: Taxonomic revision of the genus Amphritea supported by genomic and in silico chemotaxonomic analyses, and the proposal of Aliamphritea gen. nov
Source: PLoS One. 2022 Aug 10;17(8):e0271174. doi: 10.1371/journal.pone.0271174 (PMC9365125; doi:10.1371/journal.pone.0271174)
Supplement: S2 Table — +: used, -: not used. (PDF) [file pone.0271174.s013.pdf]

Table S2. List of other *Oceanospirillaceae* genomes used for genome taxonomy of *Aliamphritea* and *Amphritea*

Strain	Accession number	MLSA	AAI
Neptuniibater caesariensis MED92^T	GCF_000153345.1	+	+
Neptuniibacter marinus LFT 1.8^T	GCF_001597735.1	+	+
Neptuniibacter pectenicola ATR 1.1^T	GCF_001597725.1	+	+
Neptunomonas antarctica DSM 22306^T	GCF_900156635.1	+	+
Neptunomonas phycophila Scap09	GCF_013394205.1	+	+
Marinobacterium aestuarii ST58- 10^T	GCF_001651805.1	+	+
Marinobacterium georgiense IC961^T	GCF_017310015.1	+	+
Marinobacterium litorale DSM 23545^T	GCF_000428985.1	+	+
Marinobacterium mangrovicola DSM 27697^T	GCF_004339595.1	+	+
Nitrincola tapanii MEB 193^T	GCF_008368715.1	+	+
Nitrioncola lacisaponensis 4CA^T	GCF_000691225.1	+	+
Nitrincola tibetensis xg18^T	GCF_003284585.1	+	+
Marinomonas mediterranea MMB-1^T	GCF_000192865.1	+	-
Marinomonas posidonica IVIA-Po-181^T	GCF_000214215.1	+	-
Marinomonas arctica BSI20414	GCF_014623465.1	+	-
Oceanospirillum beijerinckii DSM 7166^T	GCF_000422425.1	+	-
Oceanospirillum maris DSM 6286^T	GCF_000422865.1	+	-
Oceanospirillum multiglobuliferum ATCC 33336^T	GCF_900167095.1	+	-

+: used, -: not used.
