# Supplementary material for: Taxonomic revision of the genus Amphritea supported by genomic and in silico chemotaxonomic analyses, and the proposal of Aliamphritea gen. nov
Source: PLoS One. 2022 Aug 10;17(8):e0271174. doi: 10.1371/journal.pone.0271174 (PMC9365125; doi:10.1371/journal.pone.0271174)
Supplement: S3 Table — PG, phosphatidylglycerol; PE, phosphatidylethanolamine; DPG, diphosphatidylglycerol; GPL: glycophospholipid. nd: not determined. (PDF) [file pone.0271174.s014.pdf]

Table S3. Fatty acid, isoprenoid quinone and polar lipid profile of previously reported *Amphritea* and *Aliamphritea* species

	Aliamphritea ceti	*Aliamphritea spongicola*	*Amphritea atlantica*	*Amphritea japonica*	*Amphritea balenae*	*Amphritea opalescens*	*Amphritea pacifica*
**Predominant FA**							
**$C_{16:0}$**	20%	20%	30%	20%	10%	20%	20%
**$C_{16:1}\omega 7c$ and/or $C_{16:1}\omega 6c$**	50%	40%	40%	40%	50%	40%	40%
**$C_{18:1}\omega 7c$ and/or $C_{18:1}\omega 6c$**	30%	30%	20%	40%	30%	20%	20%
**Hydroxy FA**							
**$C_{10:0}$ 3-OH**	<10%	<10%	<10%	<10%	<10%	<10%	<10%
**$C_{12:1}$ 3-OH**	<10%	<10%	<10%	<10%	<10%	-	-
**Major lipid class**							
**PG**	+	+	+	+	+	+	+
**PE**	+	+	+	+	+	+	+
**DPG**	nd	nd	nd	nd	nd	+	nd
**GPL**	nd	+	nd	nd	nd	nd	nd
**Isoprenoid quinone**	Q-8	Q-8	nd	Q-8	Q-8	Q-8	Q-8
**References**	[3]	[4]	[1]	[2]	[2]	[5]	[6]
