# Supplementary material for: Taxonomic revision of the genus Amphritea supported by genomic and in silico chemotaxonomic analyses, and the proposal of Aliamphritea gen. nov
Source: PLoS One. 2022 Aug 10;17(8):e0271174. doi: 10.1371/journal.pone.0271174 (PMC9365125; doi:10.1371/journal.pone.0271174)
Supplement: S4 Table — (PDF) [file pone.0271174.s015.pdf]

Table S4. Results of 3D-structure prediction of Des1-4 by Phyre2

	PDB Molecule	Template	Confidence	Coverage	Identity
Aliamphritea hakodatensis Des1	Stearoyl-CoA desaturase	c4zyoA	99.9%	77%	12%
Aliamphritea ceti Des1	Stearoyl-CoA desaturase	c4zyoA	99.9%	81%	12%
Aliamphritea spongicola Des1	Stearoyl-CoA desaturase	c4ymkA	99.8%	78%	11%
Aliamphritea hakodatensis Des2	Stearoyl-CoA desaturase	c4zyoA	99.9%	75%	12%
Aliamphritea ceti Des2	Stearoyl-CoA desaturase	c4zyoA	99.9%	75%	11%
Aliamphritea spongicola Des2	Stearoyl-CoA desaturase	c4zyoA	99.9%	75%	13%
Aliamphritea hakodatensis Des3	Stearoyl-CoA desaturase	c4zyoA	99.9%	84%	11%
Aliamphritea ceti Des3	Stearoyl-CoA desaturase	c4ymkA	99.9%	77%	11%
Aliamphritea spongicola Des3	Stearoyl-CoA desaturase	c4zyoA	99.8%	76%	13%
Amphritea balenae Des4	Stearoyl-CoA desaturase	c4ymkA	99.9%	87%	15%
Amphritea japonica Des4	Stearoyl-CoA desaturase	c4ymkA	99.9%	85%	13%
