# Supplementary material for: Taxonomic revision of the genus Amphritea supported by genomic and in silico chemotaxonomic analyses, and the proposal of Aliamphritea gen. nov
Source: PLoS One. 2022 Aug 10;17(8):e0271174. doi: 10.1371/journal.pone.0271174 (PMC9365125; doi:10.1371/journal.pone.0271174)
Supplement: S5 Table — +: genes presence; -: absence. (PDF) [file pone.0271174.s016.pdf]

Table S5. PG, PE and DPG associated genes composition of each strain

	Aliamphritea hakodatensis	*Aliamphritea ceti*	*Aliamphritea spongicola*	*Amphritea atlantica*	*Amphritea japonica*	*Amphritea balenae*	*Amphritea opalescens*	*Amphritea pacifica*
plsX	+	+	+	+	+	+	+	+
plsY	+	+	+	+	+	+	+	+
plsC	+	+	+	+	+	+	+	+
cdsA	+	+	+	+	+	+	+	+
pssA	+	+	+	+	+	+	+	+
psd	+	+	+	+	+	+	+	+
pgsA	+	+	+	+	+	+	+	+
pgpA	+	+	+	+	+	+	+	+
cls	-	-	-	+	+	-	+	+

+: genes found; -: not found.
