# Supplementary material for: Taxonomic revision of the genus Amphritea supported by genomic and in silico chemotaxonomic analyses, and the proposal of Aliamphritea gen. nov
Source: PLoS One. 2022 Aug 10;17(8):e0271174. doi: 10.1371/journal.pone.0271174 (PMC9365125; doi:10.1371/journal.pone.0271174)
Supplement: Attachment — Submitted filename: Response_letter_PONE-D-21-39007.docx [file pone.0271174.s017.docx]

Dear Professor Vaz-Moreira, academic editor, and reviewers,

We appreciate editor and reviewers for constructive suggestions. We improved the manuscript PONE-D-21-39007 according to the reviewers’ comments. Responses for specific comments are described as follows. All changes were highlighted in yellow. Also, we noticed that tables were in graphic object form, so we changed them into editable objects.

Reviewer: 1

|  | Reviewer’s suggestion | Response |
| --- | --- | --- |
| 1 | Analysis of the bacterial cell fatty acids is necessary | Thank you for the suggestion. We understand the importance of FAME profiling but no budget for performing the FAME cannot allow us to have the results. We could not find good facility to conduct the FAME on time. Instead of the FAME profiling, in depth in silico chemotaxonomy prediction using the complete genome sequences of Amphritea and PT3 was performed, and we conclude C16:0, C16:1, and C18:1 are major FAME of PT3 similar to the other described Amphritea spp. We also did careful prediction that PT3 could produce Q-8, and PE and PG as well. We hope this in silico chemotaxonomy contribute new and deeper knowledge on how and which fatty acids are produced by Amphritea and Aliamphritea spp. |
| 2 | L36: “sp., nov.” -> “sp. nov.” | Thank you for the suggestion. We did correct it. |
| 3 | L80: What does the abbreviation “ASW” mean? | We corrected “ASW” into “artificial seawater”. |
| 4 | L95: Delete this word “(BD)” | We corrected it. |
| 5 | L111: What does the abbreviation “DDH” mean? | “DDH” stands for “DNA-DNA hybridization”. We added this information. |
| 6 | L119: These gene (*recA, mreB, rpoA,* and *topA*) different from the abstract is described. | Thank you so much for the comment. We corrected. |
| 7 | L141: JCM1668^T^ -> JCM 16668^T^ | We corrected it. |
| 8 | L163: JAMM 1525^T^ -> JCM 14781^T^ | We corrected it. |
| 9 | L178: Please change the following reference "(Lee et al., 2018)" to a number. | We corrected it. |
| 10 | L184: The minimum and maximum values in Fig. 2 are different. | Thank you for the comment. We checked the ANI values in Fig. 2 and corrected the numbers. |
| 11 | L217-238: I don't know where the explanations in Fig5, Fig.6, and Table2 are separated. | Thank you for the comment. The explanation for Fig 6. is directly after the figure title, starting from “Dots closer to the diagonal line represents”. We added explanation for Fig7 about the scale bar. As for Table2, we ask the correction in the production process. |

Reviewer: 2

|  | Reviewer’s suggestion | Response |
| --- | --- | --- |
| 1 | The type species of the novel genus is demonstrated as *Aliamphritea ceti*, hence, *Aliamphritea hakodatensis* could not described as gen. nov. sp. nov. but sp. nov. only | Thank you so much the suggestion. We corrected the description part according to the suggestion. |
| 2 | The title could be changed as “*Aliamphritea* gen. nov. by genome based reclassification of the genus *Amphritea* and reclassification of *Amphritea spongicola* and *Amphritea ceti* as *Aliamphritea spongicola* comb. nov. and *Aliamphritea ceti* comb. nov. and *Aliamphritea hakodatensis* sp. nov.” or “*Aliamphritea ceti* gen. nov. comb. nov. and *Aliamphritea spongicola* comb. nov. via genome based reclassification of the genus *Amphritea* and *Aliamphritea hakodatensis* sp. nov.”. | Thank you so much for the suggestion. We changed the title to “*Aliamphritea* gen. nov. by genome based reclassification of the genus *Amphritea* and reclassification of *Amphritea spongicola* and *Amphritea ceti* as *Aliamphritea spongicola* comb. nov. and *Aliamphritea ceti* comb. nov. and *Aliamphritea hakodatensis* sp. nov.” |
| 3 | Description of the genus looks not fully adequate. In case of temperature, described as “mesotrophic” seems better and pH and salt response, basic components of the fatty acids or polar lipids, and respiratory quinone types could be included but DNA hydrolysis looks not. Additionally, parallel description of the isolation sources could be summarized as “marine animals” or describing as “marine animal related sources such as~” seems better. | Thank you for the suggestion. We improved the description. |
| 4 | Does there no changes in the description of two combined species compared to former description? Please check carefully and change. | Thank you so much for you suggestion. Of course, phenotype results were different laboratory by laboratory. This is common sense, so we did not mention the differences. Only results by our laboratory were described. |
| 5 | About the genomic features: The authors described about PHB synthesis related genes in the core genes in two genera but nothing about function of core genes in each genus. Difference of genomic features could be a reason for dividing and important in the recognition of ecological role of two genera. Please including extensive analysis on genomic features of the two genera. | Thank you for the suggestion. We added extended analysis of the core genomes of each genus. |
| 6 | Fig. 1 : Please include compared base no. and including descriptions on size marker and bootstrap values in the figure title. | Thank you for the suggestion. We improved. |
| 7 | Fig. 2 : Actually it’s not very important and it could be combined with Fig. 3. Move to supplementary materials or remove but including values in the Fig. 3. And ANI values against type species could be included in the Table 1.  Fig. 3 : As mentioned above, ANI values could be included in the half of the table format. | Thank you for the suggestions. We moved Fig 2 to the supporting information as Fig S1. |
| 8 | Fig. 5 : Require explanation on color level. | Thank you for the suggestion. We added the following explanation for Fig 5, “The darker colored areas of the bars belong to one of the three bins; Core, *Aliamphritea* specific or *Amphritea* specific.” |
| 9 | Fig. 6 : Considering the (C) and (D), (E) could be looks like as (A) or (B). More explanation is required. | Thank you for the suggestion. We added some information for (E). |
| 10 | Table 1 could be moved to supplementary materials. | We moved it. |
| 11 | Table 2 : Please include other species also and explain “I” is what meaning?  Additionally, genus level comparison is required at least genera included in the Fig. 3 for genus suggestion. | Thank you for the suggestion. We added phenotype results of the two *Amphritea* species from previous studies. We also provide genus phenotype comparison table. |
| 12 | Line 26: among -> above | Thank you for the suggestion. We corrected it. |
| 13 | Line 28 : Who’s AAI values? It’s not clearly described. | Thank you for the comment. In this sentence, we are discussing AAI values of PT3^T^ against *Amphritea* species. We changed the sentence to “Furthermore, the AAI values of PT3^T^ against other *Amphritea* species were on the reported genus delineation boundary (64-67%).” |
| 14 | Lines 89-90: What kind of method was used for distance calculation? It should be included with options. In this term, Lines 156-158 should be moved to “materials and method” part. | Thank you for the suggestion. The method was Kimura 2-parameter model, and we added this information. We also moved it. |
| 15 | Line 156: What is the K2+G+I model? Please described fully. | Thank you for the suggestion. K2+G+I is an abbreviation for Kimura 2-parameter with gamma distribution and invariant site. We added this information in the description of Fig 1. |
| 16 | Line 185: Please provided original article as reference. | Thank you for the suggestion. We added an explanation for Table 1 which includes the original article that sequenced draft genome for *A.opalescens* and *A.pacifica*. |
