# Supplementary material for: Taxonomic revision of the genus Amphritea supported by genomic and in silico chemotaxonomic analyses, and the proposal of Aliamphritea gen. nov
Source: PLoS One. 2022 Aug 10;17(8):e0271174. doi: 10.1371/journal.pone.0271174 (PMC9365125; doi:10.1371/journal.pone.0271174)
Supplement: Attachment — Submitted filename: Response_letter_May7_Yamano_D-21-39007R2.docx [file pone.0271174.s018.docx]

Dear Professor Yurchenko, the academic editor, and reviewers,

We appreciate editor and reviewers for constructive suggestions. We improved the manuscript according to the reviewers’ comments. Responses for specific comments are described as follows. All changes were highlighted in yellow.

Reviewer: 1

|  | Reviewer’s suggestion | Response |
| --- | --- | --- |
| 1 | The idea of "in silico chemotaxonomy" is necessary as a new chemotaxonomy,  and the chemotaxonomic information in this manuscript is described.  However, it is necessary to discuss whether it can be used in a wide  range of bacterial species. | Thank you very much for the positive comments. We did try to estimate all possible pathways commonly used in traditional chemotaxonomy for Amphritea and Aliamphritea. These pathways are very likely to be similar to E. coli, so this methodology could easily be applied to the Gammaproteobacterial group. We also tried to estimate PUFA and branched FA pathways, so this methodology in silico chemotaxonomy could be applied all bacterial taxa, but only qualitatively for now. We are also trying to compare the 3D structure of key enzymes which are responsible for regulating the carbon chain of FA, if these approaches are successful, we might estimate the length of FA. We added a discussion about this at the end of the “In silico chemical taxonomy” section. |
| 2 | P15-P24. There are many misspellings, so please reconfirm. | Thank you for the comment. We have corrected them. |
| 3 | Table 1-3. Is it possible to summarize the order of species with *Aliamphritea* and *Amphritea*? Table 1-3 wants the order of the genera to be the same | We rearranged the species order. |
| 4 | Figures 6, 7, 8 and 9 should be moved to supplementally | We moved Figs. 7-9 to supporting information, but Fig. 6 was not moved because it is necessary to show the estimated fatty acid synthetic pathway in PT3T and its relatives. |

Reviewer: 2

|  | Reviewer’s suggestion | Response |
| --- | --- | --- |
| 1 | As recommended, title was changed but same matters are remained in  the abstract and main body of the MS.  i. Lines 34-41 also should be changed in accordance with the title. It can be suggested as follows; Depending on genome-based approaches, *Aliamphritea* gen. nov. was proposed with reclassification of the genus *Amphritea* and *Aliamphritea ceti* comb. nov. RA1^T^ (=KCTC 42154^T^ =NBRC 110551^T^), *Aliamphritea spongicola* comb. nov MEBiC05461T (=KCCM 42943T =JCM 16668T), and *Aliamphritea hakodatensis* sp. nov. PT3^T^(= JCM 34607^T^=KCTC 82591T). were suggested.  ii. Lines 58-63 : Please include the contents about dividing genus  *Amphritea* into two genera.  iii. Conclusion part also should be changed. | Thank you for the suggestion. We added reclassification of Amphritea in the Abstract and introduction. We also modified the conclusion. |
| 2 | Line 445 : remove “comb. nov.” | We have removed it. |
| 3 | Line 447 : remove “gen. nov.” | We have removed it. |
| 4 | Lines 450-451 and 457-458 : Genome of these species were newly reported, hence, including genome accession number is required. | We have added them in the description. |
| 5 | Line 482 : Provided number was not accession, please correcting it. | We have added them in the description. |
| 6 | lines 256-385 seems not important part of the analysis of genome repertories, these informs could be obtained by chemical analysis but ecological or physiological importance were unclear. | This part described the results and discussion of in silico chemical taxonomy for PT3T, which means it’s one of the important parts of this study. We determined PT3T chemical taxonomy profile by comparing experimental chemical taxonomy data with the responsible gene sets among Amphritea spp. We also put subtitle to indicate the importance of this part. Ecological and physiological importance was not mentioned here. |
| 7 | strengthen the contents like in lines 202-237 (difference between two genera) seems better for readers. | We described core (same) and unique gene sets between Amphritea and Aliamphritea. To help readers better understand, we used the genus name in this part. |
| 8 | Figs 5-6 : Could be moved to supplementary. | We have moved Fig 6 to the supplementary section. |
| 9 | Figs 7-9 : Moved to supplementary or simplified by bar plot type. | We have moved Figs 7-9 to the supplementary section. |
| 10 | Fig. 10 : Not very important and could be moved to supplementary. | We have moved it to the supplementary section. |
| 11 | Table 1 : If not including that of strain PT3^T^ it also moved to supplementary materials. | We have moved it to the supplementary section. |
| 12 | Table S7 should be included in main body and Table 3 : Only members in *Aliamphritea* is enough. | We have moved Table S7 to the main body. We kept the experiments results from *Amphritea* members because those results are necessary to distinguish *Aliamphritea* members from *Amphritea* members. |
| 13 | Line 47: genera -> genus | We have corrected it. |
| 14 | Lines 47-50 : Amphritea could be abbreviated as A. (eg A. japonica). | For the abbreviation of genus name, all genus name not only bacteria but also sea cucumber which PT3 isolated are started by "A”, so we decided not to use abbreviated form of genus name to prevent confusion. |
| 15 | Lines 72, 97, etc. : missing ‘,’.. | We have corrected it. |
| 16 | Lines 85 etc. : Check all PT3^T^ in the MS, many times missing T and  many times has interval between 3 and T. | We have corrected it. |
| 17 | Lines 179-182: “~ against above species~” seems enough. | We have rephrased it. Also we rearranged the order of the values. |

Reviewer: 3

|  | Reviewer’s suggestion | Response |
| --- | --- | --- |
| 1 | I understand that the manuscript title was suggested by a reviewer, but I find it very long with unnecessary details. I suggest shortening it, e.g., “Taxonomic revision of the genus Amphritea supported by genomic and chemotaxonomic analyses”. | Thank you for the suggestion. We have changed the title. |
| 2 | My major problem is lack of discussion. Although the chapter is named “  Results and Discussion”, a large part of it is merely descriptive with  very few biological inferences drawn from the results. | We added some discussions. Also, we added subtitles in the Results and Discussion, which may help the discussion become more visible to the readers. |
| 3 | I find the usage of strain names an overkill. Surely, strain names should be clearly stated in methods, but to use them every time the species are mentioned is a bit excessive. | We have corrected it. |
| 4 | Genus names should be abbreviated when used repeatedly. | We did not abbreviate genus name to avoid confusion with the host animal of the novel strain, *Apostichopus japonicus*. |
| 4 | Authors identified 1,660 gene clusters comprising 13,930 genes named “core”. They describe functions of very few of them on L208-211. Can authors elaborate on the rest of them? | We explained abundant functions in the Core. Also, we added more information about core genes encoding important enzymes for the ecology of genera *Amphritea* and *Aliamphritea.* |
| 5 | Is difference of 10% in lipid content a significant change (Table 1)?  Can those numbers be directly compared if they come from different studies? Did those studies use the same methodology? It is beyond my power to check methods in all mentioned references. | Table 1 was based on published data. Even below 10%, presence of hydroxy fatty acid was reported to be a unique feature of Amphritea. As suggested by the reviewer, our pangenome analyses revealed that those hydroxy fatty acid might be from lipidA, which means it is not such a unique feature in Gram-negative bacteria. |
| 6 | L146: Usage of chromosome 1 is not a “complete genomic sequence” as  stated in the first part of the sentence. Please correct. Why did  authors use only 1 chromosome of A. japonica? | Thank you for the suggestion. The complete genome of *A. japonica* consisted of one chromosome and a plasmid, so in differentiating between chromosome and plasmid, we used chromosome 1, but mis-used it. We have corrected it. |
| 7 | Call out for Fig. 3 appears only in the legend of Fig. 2, but nowhere  else in the manuscript. Comment on this figure in the main text. Moreover, I think Fig. 2 can be moved to supplement. | Both Figs. 2 and 3 are important data to support the proposal of Aliamphritea gen. nov. So, both figures have not been moved to the supplementary section. |
| 8 | In Fig. 3 genes specific for a clade are called “Aliamphritea” and “ Amphritea clade specific”, but in the text it is “Ceti” and “Atlantica clade specific”. Ceti and Atlantica also appears in Fig. S3. Please unify. | We have unified it into “Aliamphritea clade specific” and “Amphritea clade specific” or “Aliamphritea” and “Amphritea”. |
| 9 | I would appreciate a composite figure of all synthetic pathways, i.e., adding Fig. S2 and S6 as panels to Fig. 6, and using the color codes for presence of genes in the two lineages (as in Fig. S2 now). If this was done, Table 2 could be moved to supplement. | Thank you for the suggestion. We have made improvements. . |
| 10 | I also do not see a point in showing genes for fatty acid, phospholipids  and ubiquinone syntheses split into separate Fig. 7-9. Please combine  them (using different colors for genes of different pathway). Right now,  it seems that some genes from different pathway are overlapping (which  may not be wrong). | Thank you for constructive comments. We tried to combine Figs. 7-9, but the resulting figure was rather confusing with too much information. So, we have moved these figures to supporting information. |
| 11 | In all tables, use species names instead of numbers and arrange species  in a logical order (Aliamphritea species together, and Amphritea together). | We rearranged the order of species in a logical order. Scientific names were not abbreviated to preventing confusion with the sea cucumber name. Please see our reply in response 4. |
| 12 | L481-482: Accession numbers are not accessible. Also add corresponding accessions to supplementary tables even if they come from this study. | We have described them in the descriptions. |
| 13 | I think tables S3 and S4 can be merged into one listing which species  were used for which analyses. Why not all species used for MLSA were  used also for AAI calculations? | We have merged two tables into one. Some of the relatively phylogenetically distant genera were not used for AAI calculation to avoid the heatmap becoming too big. |
| 14 | Fig. S1 in its current form is rather confusing. I have no idea how the numbers correspond to the species names. Can this be presented as a table? | We changed the figure into a matrix similar to Fig. 2. |
| 15 | Fig. S2 and S4 are not mentioned in the manuscript text. Please add call outs at appropriate places. | We have mentioned both of them in our new manuscript. |
| 16 | Lines 72, 97, etc. : missing ‘,’.. | We have added commas. |
| 17 | Lines 85 etc. : Check all PT3T in the MS, many times missing T and  many times has interval between 3 and T. | We have corrected it. |
| 18 | Lines 179-182: “~ against above species~” seems enough. | We have rephrased it. Also we have rearranged the order of the values. |
| 19 | L45: Oceanosprillales | We have corrected it to *Oceanospirillales*. |
| 20 | L97: missing comma | We have added a comma. |
| 21 | L120 and L125: Ocenospirillaceae | We have corrected it. |
| 22 | L176: missing genus names for A. opalenscence | We have added it. |
| 23 | L224-225 and 419-421: incomplete sentences | We have corrected it. |
| 24 | L273 and Table 1: Inconsistent usage of e.g. C16:0 as subscript with other parts of the text. | We have corrected it. |
| 25 | Fig. 6: cis-3-decanoyl-ACP instead of cis-3-decenoyl-ACP | We have corrected it. |
| 26 | L286-287: The last sentence of the paragraph is rather confusing. Rephase: “No genes responsible for the synthesis of ornithine-containing lipids were found in PT3 or any other Amphritea spp.” | Thank you for the suggestion. We have corrected it. |
| 27 | Please read the whole manuscript carefully and fix typos, inconsistences, formatting, etc. | We have checked the manuscript as you suggested. |
| 28 | Abbreviations ANI, DDH, and AAI are not explained in the abstract. Please add their explanation. | We have added the explanations in the abstract. |
| 29 | Methods lack primer sequences that were used for PCR and/or sequencing. | We have added primer information. |
| 30 | Results and Discussion would hugely benefit from splitting the text to subchapters and adding appropriate titles. | Thank you for the suggestion. We have added titles for each section of the Results and Discussion. |
| 31 | L109-110: Include accession numbers for retrieved genome from NCBI. | We have added them in the descriptions. |
| 32 | L148-149: I believe that authors searched for enzymes of fatty acid, polar lipid, and isoprenoid quinone synthesis. The title should be corrected. | Yes, we searched for enzymes responsible for each synthesis to predict the fatty acid, polar lipid and isoprenoid quinone profiles. |
| 33 | L152: What does “were mined from the genome sequences” mean? Proper description of tools and their parameters are necessary. Was BLAST or HMMER used? What version of the program? What was the Evalue cutoff threshold? | We have added an explanation for this. |
| 34 | L185: If I correctly understand Fig. 2, the 93.7% corresponds to comparison of PT3 and A. spongicola, while 86.9% to PT3 and A. ceti. It is switched in the text. | Thank you for finding this. The strain was switched as you mentioned, and we have corrected it. |
| 35 | L231: Similarly to one of my previous comments, genes do not encode putrescine. Please rephrase. | We have corrected this part to “genes encoding putrescine--pyruvate aminotransferase”. |
| 36 | L295: Similarly, why is Phyre2 not mentioned in methods? | We have added information about Phyre2 in the methods. |
| 37 | L267 and 311: Did authors mean linear FA by “straight-chained”? | Yes, we meant linear FA. We have changed “straight-chained” in L267 and 311to “linear” in the manuscript. |
| 38 | L315: What is meant by “homogenous to”? Is it meant that genes of the  core set in PT3 are homologous to other mentioned species? | We have deleted “particularly homogenous to A.ceti and a.spongicola” part to avoid confusion. |
| 39 | Improve visualization of Fig. 5, right now it appears fuzzy. | We have improved it. |
| 40 | What is “OF test” in Table 3? Also, “N” and “O” from the same row are  not explained. | We have added explanation about OF test and “N” and “O”. |

Reviewer: 4

|  | Reviewer’s suggestion | Response |
| --- | --- | --- |
| 1 | The chemotaxonomic characteristics summarized in Table 1 represents  published data and is therefore not original, so I propose to move it to  supplementary data. | We have moved it to supporting information. |
| 2 | Table 1: marking the species with names instead of numbers would make the table more readable. | We have marked it with species names. |
| 3 | I suggest improving the discussion, which is not visible between the results. | We added some discussion. Also, we added subtitles in the Results and Discussion, which may help the discussion become more visible. |
| 4 | Unify the naming of the new species. It is recognized as PT3 in Fig 4., and Fig.7; Aliamphritea hakodatensis in Fig.3, and both names are used in Fig. 1 and Fig. 2. | We have unified the naming of the new strain. In the main text, we use PT3^T^ and in tables and figures, we use *Aliamphritea hakodatensis* PT3^T^ or *Aliamphritea hakodatensis*. |
| 5 | Line 267: straight-chain change to linear-chain. | We have changed “straight-chained” to “linear” in the manuscript. |
| 6 | Fig S2. Predicted polyamine metabolism pathways in Aliamphritea and Amphritea: change to “Predicted polyamine metabolism pathways in  Aliamphritea and Amphritea species.” | We have changed it. |
| 7 | Fig S6. Predicted Q-8 synthetic pathway in Amphritea and Aliamphritea:  change to “Predicted Q-8 synthetic pathway in Amphritea and  Aliamphritea species. “ | We have corrected the legend of Fig S6. |
| 8 | Accession numbers of genes originated from this study should be added  into all tables instead of “in this study”. | We have added the number in the descriptions. |
| 9 | Table S2. Genomic properties of Aliamphritea and Amphritea species:see previous point. Change Alaimphritea to Aliamphritea in the first two rows. | We have corrected it. |
| 10 | Table S3. List of genomes used for AAI calculation: see the point 8. | We have changed this table listing genomes that were downloaded. |
| 11 | Table S7. Phenotypic characteristics of *Aliamphritea* and genus level comparison within the family *Oceanospirillaceae*: *Oeanospirillum* change to *Oceanospirillum* | We have corrected it. |
| 12 | Table 1 : If not including that of strain PT3^T^ it also moved to supplementary materials. | We have moved it to supporting information. |
