# Supplementary material for: Taxonomic revision of the genus Amphritea supported by genomic and in silico chemotaxonomic analyses, and the proposal of Aliamphritea gen. nov
Source: PLoS One. 2022 Aug 10;17(8):e0271174. doi: 10.1371/journal.pone.0271174 (PMC9365125; doi:10.1371/journal.pone.0271174)
Supplement: Attachment — Submitted filename: Response_letter_14June22.docx [file pone.0271174.s019.docx]

Dear editor and reviewers,

We appreciate editor and reviewers for constructive suggestions. We improved the manuscript according to the reviewers’ comments. Responses for specific comments are described as follows. All changes were highlighted in yellow.

Reviewer: 2

|  | Reviewer’s suggestion | Response |
| --- | --- | --- |
| 1 | Divide the results into two parts, one for basic reclassification and  another for further works on genomic contents.  In the MS, Aliamphritea mentioned before suggestion of the novel  genus, it makes confusion. Hence, suggestion of the genus Aliamphritea  based on phylogenetic and OGRIs results conducted first. Then, describe  genome based analysis results. | According to reviewer’s suggestion, results and discussion part was re-constructed to propose Aliamphritea gen. nov. based on 1) phylogenetic, 2) genetic and 3) phenotypic cohesions. |
| 2 | After suggestion of the genus name, ‘Aliamphritea Core” or “  Amphritea Core” makes no problems. If not, presented as “Aliamphritea  core -> ceti group core & Amphritea core -> atlantica group core” is  adequate. | Corrected accordingly. |
| 3 | Additionally, “Core” including Amphritea and Aliamphritea should be changed to “Common”. | In genome comparison, terms of core, pan, and unique are commonly used, so we would like to follow these descriptions in this manuscript. |
| 4 | If suggesting novel genus at the first half of the Results part, phenotypic comparison between species could be reduced to species in the novel genus. As it say, Table 2 composed with first 3 species is enough. Table of this time could be given as supplementary. Of course, discussion part (lines 451-466) should be amended. | Corrected accordingly. |
| 5 | Additional requirements for suggestion of novel genus: For suggestion of novel genus, genus level comparison should be given. In this case, including all genera in the family is normal process. However, Oceanospirillaceae is too big, hence, at least, compare with phylogenetically close genera such as Amphritea, Corallomonas, Neptuniibacter, Neptunomonas, Pontibaculum, Profundimonas, and Oceanispirillum is recommended. In this case, table includes basic features described in the genus is enough. | Corrected accordingly. |
| 6 | Table S1 and S2 could be combined with all information. These two are actually overlap but inform given each is not enough. | Thank you for the suggestions. After consideration, both tables were improved in title and construction. |
| 7 | Fig. 5 : For demonstrate author’s suggestion, more plots including intra groups (atlantica vs. opalescens, japonica vs. balenae) should be given. Additionally, if evolution by inversion is proved, Fig. S3 should be moved to main text and provided by combined with Fig. 5. | Unfortunately, complete genome sequences of the other Amphritea species (opalescens, balenae, pacifica) were not available currently. So, we could not change these parts. |
| 8 | Fig. 6 : Genes present or not depend on species or group should be given. At present general pathway only. | Only core genes were shown. |
| 9 | 16S rRNA gene based phylogeny (Fig. 1): Trimming inform and number of compared base should be given. | According to the suggestion, that information was provided. |
| 10 | Line 192: Need some discussion about this. Why ceti group has bigger genome? | According to the suggestion, we added some discussion in the section “Pan and core genome analysis”. |
| 11 | Title of Fig. 3: Of course, gene sequences from genome, it’s not genome tree. Hence, line 218 is not adequate. | We changed this sentence to “List of strains and their assembly accession is provided in Table S2”. |
| 12 | Lines 26 and 120: (in silico DDH) -> (isDDH) | We would like to use common description for in silico DDH, so it was unlikely to be abbreviated. |
| 13 | Lines 33-34: (PT3T, A. ceti RA1T and A. spongicola MEBiC05461T) is enough | Corrected accordingly. |
| 14 | Lines 37-39: change like as this “~ sp. nov. (type strain RA1T =  KCTC ~) | Corrected accordingly. |
| 15 | Line 106: manufactureR | Corrected accordingly. |
| 16 | Line 114: this is not actual accession number but “assembly accession  ” number. Pls change. | Corrected accordingly. |
| 17 | Line 120: Average “NUCLEOTIDE” Identity | Corrected accordingly. |
| 18 | Line 357: Des4 is not common, hence, need to rephrase. | We rephrased it. |
| 19 | Line 482: 42943T), RESPECTIVELY. | Corrected accordingly. |
| 20 | Line 482: novel genus -> genus Aliamphritea | Corrected accordingly. |
| 21 | Line 483: remove “gen. nov.” | Corrected accordingly. |
| 22 | Line 498: Remove strain name, species name is enough in here. | Corrected accordingly. |
| 23 | Lines 501 & 511: remove “,” after “et al.” | We removed it. |
| 24 | Line 521: insert “and: after “cream” | We added it. |

Reviewer: 3

|  | Reviewer’s suggestion | Response |
| --- | --- | --- |
| 1 | I suggested to remove strain names from the manuscript (except for  Methods). Authors indeed did so but starting by subchapter “Pan and core  genome analysis”, previous subchapters still contain strain names. | Corrected accordingly as possible we can. |
| 2 | Me and reviewer #2 suggested to abbreviate species names (e.g., Amphritea to A. and Aliamphritea to Al.), but authors did not comply, because it could be confused with sea cucumber Apostichopus japonicus. I still think that abbreviating bacterial names, while keeping sea cucumber in full, would increase clarity of the text. | Corrected accordingly. |
| 3 | One of the sentences that was incomplete remained incomplete (L464-466 now). | We corrected the sentence. |
| 4 | Species names were not added to the tables in main text. Tables would  be much clearer with them instead of numbers. | We added species name in the main text |
| 4 | Unify writing of thousands (with or without comma) throughout the text. | Corrected accordingly. |
| 5 | L97: rephrase -> “were calculated using the K2 model in MEGAX as well.” | Corrected accordingly. |
| 6 | L130: Table S2 is mentioned before Table S1 (L192), please correct. | Corrected accordingly. |
| 7 | L162-164: rephrase -> “3D-structure of FA desaturase encoding genes from some of the strains was predicted by Phyre2 [27].” | Corrected accordingly. |
| 8 | L176: typo “linages” -> “lineages” | Corrected accordingly. |
| 9 | L180: correct “which IS frequently used” | Corrected accordingly. |
| 10 | L198: What is “Formula2”? | We added explanation about this in methods section. |
| 11 | L198-199: Where are DDH values shown? Is there some figure or table?  Refer to it please. | There are no figures or tables for DDH values. We just describe the values in body. |
| 12 | L207: According to Fig. 2, AAI value for PT3 and A. atlantica is 64.6%, so this number should be shown in the range. | Thank you so much for notification. We corrected the number. |
| 13 | L228-229: “3-hydrokyalkanoate” -> “3-hydroXyalkanoate” | We corrected it. |
| 14 | L264: Can authors unify the use of “-aminobutyrate” in text and “4-  aminobutanoate” in Fig. S2? | We unified into 4-aminobutanoate. |
| 15 | L276: Call out of Fig. 4 is not appropriate here, there are no  particular genes shown in this figure. | We removed (Fig 4) in this part. |
| 16 | L320 and L323: I believe it should be “comprising” or “making up”  instead of “consisting”. | We rephrased them as so. |
| 17 | L359-360: “found from” -> “found in” | Corrected accordingly. |
| 18 | L381: Please check call outs for figure and table here, it does not  look correct. | Thank you. We checked, and change the call outs to (Fig 6, Table1). |
| 19 | L404-405: “which is responsible for octaprenyl-diphosphate synthesis”  repeats information from two lines above. | We removed “which is responsible for octaprenyl-diphosphate synthesis”. |
| 20 | L406: missing comma after ubiJ | We added it. |
| 21 | L406: These essential genes are not present in the scheme in Fig. S9.  Can authors describe their function at least in the text? | We added brief explanation for the proteins coded by these three genes. |
| 22 | L411: Fig. S8 is mentioned after Figs. S9 (L401) and S10 (L408),  please correct. | We corrected it. |
| 23 | L423: belonged -> belonging | We corrected it. |
| 24 | L438: “PT3Twas” – missing space | We added space. |
| 25 | L440, 443, Table 2: Unify “Tween80”, “tween 80”, “Tween 80”. | We unified them to Tween 80. |
| 26 | L447: I believe that authors wanted to say “diagnostic features […] differing from other genera were found”. If not, rewrite to make it  clear. | Yes, that is what we were trying to say. We changed this sentence like you suggested. |
| 27 | L484-485: Move accession numbers to Methods, there is no need for them in Conclusions. | We moved this information to methods. |
| 28 | Fig. 3: Remove underscores from and add italics to species names. | We improved it. |
| 29 | Fig. S3: Text of Amphritea species is fuzzy and underlined. Please  correct. | We improved it. |
| 30 | Fig. S4, S7, and S8: Can authors improve the quality of these figures? | We improved quality of the two figures. |
| 31 | Table 2: *1 and *2 should be presented as superscripts. | We corrected them to superscripts. |
| 32 | Table S3: *1 and *2 are not present in the table, but are explained  below the table. Please, correct. | We corrected it. |
| 33 | Table S4: Typos in species names A. balenae and A. japonica. | We corrected it. |
| 34 | Table S5: Typo in Al. ceti. | We corrected it. |
